# Improved right ventricular outflow tract function in patients with Tetralogy of Fallot after infundibular sparing compared to transventricular repair

**DOI:** 10.1186/1532-429X-17-S1-Q81

**Published:** 2015-02-03

**Authors:** Shiraz Maskatia, Mary K Olive, Nicholas A Dodd, Rajesh Krishnamurthy, Charles D Fraser, Emmett D McKenzie, James M Hammel, Shelby Kutty

**Affiliations:** 1 Pediatric Cardiology, Baylor College of Medicine, Houston, TX, USA; 2 Pediatric Radiology, Baylor College of Medicine, Houston, TX, USA; 3 Congenital Heart Surgery, Baylor College of Medicine, Houston, TX, USA; 4 Congenital Heart Surgery, University of Nebraska, Omaha, NE, USA; 5 Pediatric Cardiology, University of Nebraska, Omaha, NE, USA

## Background

The right ventricular infundibular sparing approach (RVIS) to repair tetralogy of Fallot (TOF) avoids a ventricular incision used in the transventricular (TV) approach. Recent data has demonstrated better global right ventricular systolic function in patients who underwent RVIS compared to those who had a TV repair, however the impact of surgical strategy on RV infundibular size and function is not specifically known. We hypothesized that patients repaired with the RVIS approach have improved RV outflow tract ejection fraction (RVOTEF) and have lower RV outflow tract volumes as assessed by cardiac magnetic resonance (CMR) compared with those repaired with the TV approach.

## Methods

**Figure 1:**
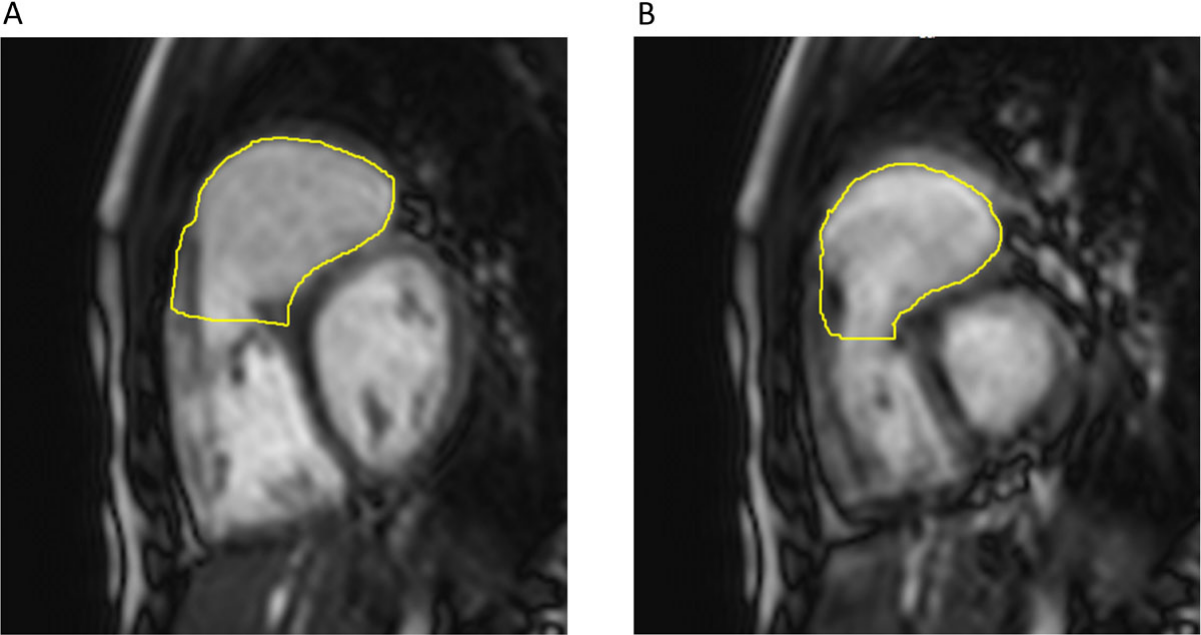
Characteristic RVOT contours at end diastole (a) and end systole (b)

A cohort of 30 TOF patients with RVIS repair and follow up CMR was identified, and compared with 30 age-matched TOF patients who had TV repair at a collaborating institution. Minor heterogeneity existed in the methods used to relieve RVOT obstruction, as efforts were made to spare the pulmonary valve in both groups. The primary outcome was RVOTEF. Secondary endpoints included end diastolic and end systolic volumes (RVOTEDV and RVOTESV) indexed to body surface area. The RVOT was contoured from the base of the septal band to the pulmonary valve superiorly at end diastole (Figure 1a) and at end systole (Figure 1b) on steady-state free-precession short-axis cine stack. An experienced blinded observer made all RVOT measurements on an independent workstation (CMR42, Circle CVI Inc, Calgary, Canada). Chi-Square, Student's t, Pearson correlation and Mann-Whitney tests were used as appropriate.

## Results

Sixty patients were included in the analysis; 30 underwent RVIS repair at median age of 10.8 months (IQR: 6.3-29.3) and 30 underwent TV repair at median age of 4.2 months (IQR: 2.2-7.0), (p<0.01). No patient in the TV group had an initial palliation with a systemic to pulmonary arterial shunt compared to 4 (13%) in the RVIS group (p<0.01). The median age at CMR was 8.4 years (IQR: 5.6-13.8) in the RVIS group and 7.9 years (IQR: 3.4-12.0) in the TV group (p=0.40). Compared to the TV group, the RVIS group had higher RVOTEF (51± 8% vs 42 ± 12%, p<0.01), lower RVOTEDV (25 ± 8 cc/m^2^ vs 32 ± 12 cc/m^2^, p=0.02), and lower RVOTESV (12 ± 4 cc/m^2^ vs 18 ± 8 cc/m^2^, p<0.01). There was a modest correlation between RVOTEF and global RVEF (R=0.42, p<0.01).

## Conclusions

Patients who underwent RVIS repair for TOF appeared to have less dilation and improved systolic function of the RVOT compared to a matched cohort repaired using the TV approach. Favorable alteration in infundibular characteristics may explain the improved RV systolic function seen with the RVIS approach. Further work is needed to determine whether these differences are predictive of clinical outcomes.

## Funding

None.

